# Screening vs. no screening for preterm delivery in low-risk singleton pregnancies: comparison by propensity score analysis

**DOI:** 10.1007/s00404-022-06882-w

**Published:** 2022-12-29

**Authors:** Athena P. Souka, Vasiliki Areti Maritsa, Makarios Eleftheriades

**Affiliations:** 1 Fetal Medicine Unit, Leto Maternity Hospital, 7-13 Mouson Str, 11524 Athens, Greece; 2 2nd Department of Obstetrics and Gynecology Aretaieio Hospital, Faculty of Medicine, National and Kapodistrian University of Athens, Athens, Greece

**Keywords:** Cervical length, Progesterone, Cerclage, Screening, Preterm delivery

## Abstract

**Purpose:**

To compare the effect of a policy of screening for spontaneous preterm delivery (SPD) by transvaginal cervical length (CL) measurement versus a no screening policy in the prevention of severe prematurity.

**Methods:**

Retrospective study on low-risk singleton pregnancies examined at 20–24 weeks. Two cohorts, one with SPD screening and the other without screening, were matched using propensity analysis to create the study groups. Women with short CL were treated with vaginal progesterone and/or cervical cerclage/pessary. The outcomes examined were SPD < 32 weeks (SPD 32) and SPD between 20 and 32 weeks (SPD 20–32).

**Results:**

Screening for SPD was associated with a significant reduction in the rate of SPD at less than 32 weeks (0.3 vs. 0.8%, *p* = 0.001 in the screened and no screened pregnancies, respectively) and in the rate of SPD 20–32 (0.3 vs. 0.9%, *p* = 0.005 in the screened and no screened pregnancies, respectively). After adjusting for maternal age, parity, body mass index, smoking and mode of conception, the screening group had significantly lower hazard for SPD 20–32 (HR = 0.36, 95% CI: 0.18–0.75, *p* = 0.006) and SPD32 (HR = 0.39, 95% CI: 0.19–0.82, *p* = 0.013).

**Conclusion:**

Screening for SPD by transvaginal CL measurement in mid-pregnancy may reduce the incidence of severe prematurity in low-risk singleton pregnancies.

## What does this study add to the clinical work

**Table:** 

The study provides evidence on the value of screening for preterm delivery in low-risk singleton pregnancies by cervical length measurement at 20–24 weeks. The screened population had significantly lower chance of preterm birth less than 32 weeks compared to the not- screened population (0.3% versus 0.8% respectively).	

## Introduction

The deleterious short- and long-term effects of preterm birth on survival and quality of life are well recognized [1]. However, identifying the pregnancies that will deliver early can be challenging. The strongest screening tool to predict spontaneous preterm delivery (SPD) in both the high- and low-risk pregnancies is the transvaginal ultrasound measurement of the cervical length (CL) [2]. CL provides useful information throughout gestation but it is the mid-trimester measurement that has been primarily studied [3–12]. Mid trimester CL measurement has moderate value in predicting any SPD but is quite sensitive in predicting early and very early SPD (before 32 and 28 weeks, respectively) which is clinically very important [9]. The value of vaginal progesterone for preventing spontaneous preterm delivery in different high-risk groups has been recognized for more than a decade [13]. Furthermore vaginal progesterone appears to be safe for the developing fetus and child [14]. In addition to vaginal progesterone, other therapies such as cervical cerclage and pessary have been proposed for pregnancies at risk for SPD because of cervical shortening [15–17].

Despite the evidence of a beneficial effect of these interventions, implementation of CL measurement in the clinical practice has been reluctant. The few studies that report on the effect of routine CL measurement in mid-pregnancy have mostly had favorable results although comparisons have only been made with historic cohorts prior to screening.

We present the effect of a policy of screening compared to a policy of no screening by transvaginal CL measurement at 20–24 weeks in the prevalence of early SPD in two low-risk cohorts using propensity data analysis to account for confounding variables.

## Methods

Retrospective study on two groups of singleton pregnancies presenting for routine second trimester detailed anomaly ultrasound scan at 20 to 24 weeks in two private fetal medicine units between January 2006 and December 2015. One unit offered transvaginal ultrasound for measurement of CL to all pregnant women and the other did not. Maternal medical and obstetric history, demographic characteristics, and pregnancy outcome were retrieved from the records (Astraia software). The group with CL screening has been reported previously [18].

CL was measured transvaginally with a 9 MHz probe (Voluson E8 and GE, Voluson Expert 730, USA) as previously described [18]. Briefly, the bladder was emptied and the longitudinal plane of the cervical canal was identified using the endocervical mucosa as the guideline. No pressure was applied so that the anterior and posterior cervical lips were of similar size. Attention was paid to distinguish between the cervical canal (as marked by the endocervical mucosa) and the lower segment of the uterus. The ultrasound examinations were performed by experienced physicians.

The screening unit included the CL measurement and the calculation of risk for SPD in the report to the referring obstetrician [9]. The use of vaginal progesterone was recommended when CL was equal to or less than 15 mm. The choice of treatment (i.e., vaginal progesterone, cervical cerclage or cervical pessary) was left at the discretion of the obstetrician. In the majority of cases, vaginal progesterone was the first line of treatment, whereas cerclage or pessary were placed when there was persistent shortening despite progesterone treatment, as identified by follow-up ultrasound scans.

Women with previous spontaneous preterm delivery, second trimester miscarriage, history of cervical surgery or congenital uterine malformations were excluded from the analysis. Singleton pregnancies resulting from embryo reduction or intrauterine death of one twin were also excluded.

The study outcomes were spontaneous preterm delivery between 24 and 32 weeks (SPD32, delivery between 24 and 31 weeks and 6 days) and spontaneous preterm delivery at < 32 weeks or spontaneous miscarriage between 20 and 23 weeks (SPD20-32, delivery between 20 and 31 weeks and 6 days).

### Statistical analysis

Continuous variables were expressed as mean and standard deviation (SD) while qualitative variables were expressed as absolute and relative frequencies. For the comparison of means between two groups, Student’s *t* test was used. For the comparisons of proportions, Chi-squared tests were used. Propensity score matching was performed to generate a study cohort of matched cases undergoing screening adjusted for potential confounding. Propensity scores were estimated using logistic regression models with performance of screening as the dependent variable. Propensity score matched cohort was constructed by nearest neighbor matching of one case undergoing screening to one case not undergoing screening. A multivariate Cox proportional hazard model was conducted to determine if screening was associated with hazard for SPD < 32 and SPD 20–32. Statistical significance was set at 0.05 and analyses were conducted using SPSS statistical software (version 24.0).

## Results

The initial sample consisted of 10,133 singleton pregnancies (6,913 with and 3,220 without SPD screening). After propensity score matching, a study sample was created consisting of 3,103 cases with screening (screening group) matched with 3103 cases without screening (no screening group). Demographic characteristics of the samples before and after matching are presented in Table 1.

**Table 1:** Demographic characteristics of the SPD screening and no screening groups, before and after propensity score matching

	Initial sample		*P*	Propensity score matched sample		*P*
	(*N* = 10,133)			(*N* = 6206)		
	No screening (*N* = 3220; 31.8%)	Screening (*N* = 6913; 68.2%)		No screening (*N* = 3103; 50%)	Screening (*N* = 3103; 50%)	
Maternal age, mean (SD)	32.8 (4.3)	32.5 (4.2)	0.002+	32.7 (4.2)	32.7 (4.3)	0.765+
BMI, mean (SD)	24.5 (6.5)	23.9 (4.5)	< 0.001+	24.3 (5.3)	24.2 (5.0)	0.886+
Smoking, *N* (%)						
No	2874 (89.3)	6048 (87.5)	0.011+ +	2766 (89.1)	2720 (87.7)	0.068+ +
Yes	346 (10.7)	865 (12.5)		337 (10.9)	383 (12.3)	
Parity, *N* (%)						
0	2374 (73.7)	5013 (72.5)	0.202+ +	2267 (73.1)	2258 (72.8)	0.797+ +
≥ 1	846 (26.3)	1900 (27.5)		836 (26.9)	845 (27.2)	
Conception, *N* (%)						
Spontaneous	6761 (96.9)	3691 (92.6)	< 0.001+ +	2997 (96.6)	2997 (96.6)	1.000+ +
Assisted	214 (3.1)	295 (7.4)		106 (3.4)	106 (3.4)	

*SPD *spontaneous preterm delivery

+Student’s *t* test

+ +Pearson’s Chi-squared test

**Fig. 1:**
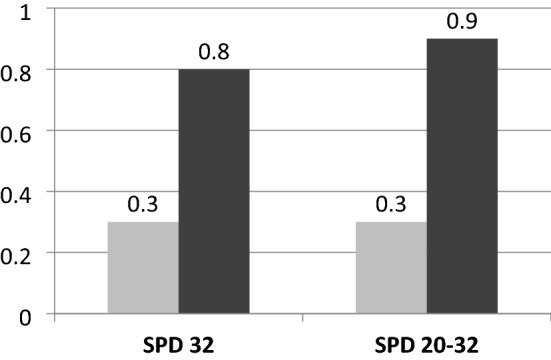
The rate of spontaneous preterm delivery < 32 weeks (SPD < 32) and spontaneous preterm delivery between 20 and 32 weeks (SPD 20–32) in the group with (light grey) and without (dark grey) screening. *AP Souka* project development, data collection, data analysis, manuscript writing, *VA Maritsa* data analysis, manuscript writing, *E Eleftheriades* data collection, manuscript editing

In the screening group, median CL was 36 mm, CL ≤ 15 mm, CL ≤ 20 mm CL ≤ 25 mm were present in 1, 3.1, and 5% of cases expectedly. Cervical cerclage was placed in 26 women. The mean gestational age at delivery was 38.4 weeks for the study sample (SD = 1.5) and was similar for cases with and without screening (*p* = 0.116). The rate of SPD32 was 0.6% in the study sample and it was significantly lower in the screening compared to the no screening group (0.3 vs. 0.8% respectively, *p* = 0.011, Table 2, Fig. 1). In addition, the rate of delivery between 20 and 32 weeks was 0.6% in the total sample and it was significantly lower in the screening group (0.3 vs. 0.9% respectively, *p* = 0.005, Table 2, Fig. 1). Using multiple Cox regression analysis and after adjusting for maternal age, parity, body mass index, smoking and mode of conception, the screening group had significantly lower hazard for SPD 20–32 (HR = 0.36, 95% CI: 0.18–0.75, *p* = 0.006, Table 3) and SPD32 (HR = 0.39, 95% CI: 0.19–0.82, *p* = 0.013, Table 4). Assisted conception was significantly associated with greater hazard for SPD 20–32 and SPD 32.

**Table 2:** Gestational age at delivery and rate of spontaneous preterm delivery < 32 weeks (SPD < 32) and spontaneous preterm delivery delivery between 20 and 32 weeks (SPD20-32) for the screening and no screening groups

	Total sample (*N* = 6206)		No screening		Screening	
	Mean	SD	Mean	SD	Mean	SD
Gestational age (weeks)	38.4	1.5	38.4	1.6	38.2	1.4
	*Ν*	%	*Ν*	%	*Ν*	%
PD < 32						
Yes	6171	99.4	3078	99.2	3093	99.7
No	35	0.6	25	0.8	10	0.3
PD < 32 and/or late miscarriage						
No	6169	99.4	3076	99.1	3093	99.7
Yes	37	0.6	27	0.9	10	0.3

**Table 3:** Hazard ratios (HR) for spontaneous preterm delivery between 20 and 32 weeks with or without screening

	HR	95.0% CI for HR		*P*
		Lower	Upper	
Group (screening vs. no screening)	0.36	0.18	0.75	0.006
Maternal age	1.07	0.99	1.15	0.102
parity (≥ 1 vs. 0)	0.50	0.21	1.22	0.128
ΒΜΙ	0.99	0.93	1.06	0.760
Smoking (yes vs. no)	2.16	0.98	4.73	0.055
Conception (assisted vs. spontaneous)	3.22	1.18	8.78	0.022

**Table 4:** Hazard ratios (HR) for spontaneous preterm delivery before 32 weeks with or without screening

	HR	95.0% CI for HR		*P*
		Lower	Upper	
Group (screening vs. no screening)	0.39	0.19	0.82	0.013
Maternal age	1.05	0.98	1.14	0.189
parity (≥ 1 vs. 0)	0.44	0.17	1.15	0.094
ΒΜΙ	0.99	0.93	1.06	0.834
Smoking (yes vs. no)	1.94	0.85	4.46	0.117
Conception (assisted vs. automatic)	3.55	1.30	9.74	0.014

## Discussion

### Main findings

This study explores the benefit of implementing universal screening for SPD by transvaginal CL measurement at the 20–24 weeks anomaly scan visit. Both the screening and the no screening groups were examined in two private fetal medicine settings at the same time period. We opted to consider only low-risk women because this is the cohort that accounts for the majority of the preterm births while, on the other hand, they receive less attention regarding their SPD risk. In addition, we included, in our outcomes, pregnancies resulting in late miscarriage that could potentially have been prevented by CL measurement. Propensity analysis was used to minimize the influence of confounding variables in the two groups. This type of analysis creates study cohorts similar to the baseline characteristics defined by the logistic regression analysis and reduces bias.

We observed a significant reduction of about 60% in the SPD32 rate and the SPD20-32 in the women that had SPD screening (Table 2, Fig. 1). This was not accompanied by a difference in the mean gestational age at delivery between the two groups which rather points to a shift of the preterm births after 32 weeks in the screened group. We also observed that assisted conception was a significant risk factor for late miscarriage as well as SPD (Tables 3, 4).

### Strengths and limitations

The strength of our study relies in the large number of subjects and the homogenous population created after the propensity analysis. More robust conclusions would be derived from a randomized trial comparing universal screening to no screening and this is the main limitation of the study. Another limitation is that the treatment for CL shortening was not uniform. However, these results probably reflect more accurately how a policy of SPD screening would work in real life conditions.

### Interpretation

In our previous study on universal SPD screening, the rate of SPD was compared before and after implementation of the policy in the same institute [18]. A population of 10,506 singleton pregnancies was assessed with a decline rate of 1.32%. High-risk women contributed only 16% of the total SPD cases. SPD at less than 34 weeks occurred in women with very short CL ≤ 15 mm in 38% of high-risk and 18% of low-risk women despite treatment. In women with CL ≤ 15 mm receiving intervention, a plateau was observed in the increase of the risk for SPD for CL 9–13 mm, whereas below 9 mm the risk increased exponentially. Following the introduction of mid-trimester CL measurement, there was a trend for a reduction in the rate of any SPD < 34 weeks of about 20% in comparison with the pre-screening period.

Likewise, Temming et al. evaluated the acceptability of a universal CL screening program, the risk factors associated with declining screening, and the subsequent delivery outcomes of women who accepted or declined screening [19]. Transvaginal CL measurement was offered at 17–23 weeks of gestation to all women with singleton pregnancies and expectant mothers could choose to opt out. The acceptance rate was high (85%) and stabilized after the first 6 months of introducing the policy of CL measurement. Patients with CL ≤ 20 mm (1.2% of the screened population) were considered to have clinically significant cervical shortening and were offered treatment. The rate of spontaneous preterm birth < 28 weeks was double in those who declined screening, although delivery at less than 34 and less than 37 weeks was not significantly different. Similarly Son et al. reported that the introduction of a universal SPD screening program in women without a history of preterm birth was associated with a reduction in the frequency of prematurity [20]. CL was evaluated at 18–24 weeks of gestation in singleton low-risk pregnancies and the SPD rates were compared before and after the implementation of the screening program. The authors report that the introduction of the program was associated with a significant decrease in the frequency of preterm birth of about 25% for SPD < 37 and SPD < 34 weeks.

In contrast, van Os et al. reported a non-significant reduction in SPD < 32 weeks and SPD < 34 weeks in low-risk singleton pregnancies identified after universal screening [21]. The authors report a very small percentage of short CL in their population probably because of methodological problems (the isthmus was included in the CL measurement).

The magnitude of the benefit in our study is higher than previously reported and higher than the reduction of SPD observed in randomized trials of progesterone versus placebo [22, 23]. Possible factors accounting for the difference is first that we examined only low-risk pregnancies and second that the median CL in the screened population is lower whereas the percentage of short CL is higher than other studies [20, 21]. Similarly, to our study, Son et al. examined singleton low-risk women but the prevalence of the short cervix, defined by the authors as ≤ 25 mm, was only 0.89% compared to 5% in our data [21]. If the sonographer exerts too much pressure to the cervix or the if the uterine isthmus is included in the measurement, the majority of the shorter and commonly softer cervices remains unnoticed. The implication is that women that may benefit from treatment are not identified and there is no benefit from the screening process. In addition, in our study, the therapeutic options in the intervention group included not only progesterone but cervical cerclage as well if CL was very short. Souka et al. have recently presented data showing that if CL is less than 9 mm at screening, cerclage is more efficient than progesterone in preventing SPD [24]. It is also possible that preventive measures such as progesterone and cerclage are more effective in low-risk pregnancies.

The finding that assisted conception pregnancies have a higher risk of preterm birth even in women without relevant history is important and confirms previous reports [25]. This increased risk may be the result of multiple procedures such as hysteroscopy that include cervical manipulation or may be inherent to the population that need fertility treatment.

Several analyses were performed regarding the cost effectiveness of the implementation of universal CL screening and treatment with vaginal progesterone to prevent SPD [26–28]. Einerson et al. compared three cervical length screening strategies in a population of women with no prior SPD: the risk-based screening and the universal screening were more effective and less costly than no screening at all [25]. Comparing between the two screening strategies, universal screening, despite costing more, resulted in being more cost effective when considering additional costs from the short- and long-term consequences of prematurity. The benefit persists even after taking into account reduced incidence of CL ≤ 20 mm at initial screening (0.83%), vaginal progesterone supplementation for women with CL ≤ 20 mm, additional ultrasound(s) for women with CL 21–24.9 mm, and the assumption that vaginal progesterone reduces the rate of preterm birth < 34 weeks of gestation by 39% if a short CL is diagnosed.

## Conclusion

Our study used propensity analysis to compare screening for SPD by mid-trimester CL measurement to no screening in low-risk singleton pregnancies and found a significant reduction of SPD32 and SPD20-32 in the magnitude of 60% in the screened group.

It is puzzling that the attitude toward universal screening for SPD has been controversial for so long, despite evidence for the efficacy of treatment. At the same time, SPD rates along with the consequential neonatal implications have remained almost unaffected by the different measures taken worldwide and are actually still increasing in developing countries. Prematurity remains the major cause of neonatal death, hence the necessity of a screening test for all women should be indisputable.

## Data Availability

All data generated in this study are available in this article.
